# Spatial suppression due to statistical regularities is driven by distractor suppression not by target activation

**DOI:** 10.3758/s13414-019-01704-9

**Published:** 2019-03-13

**Authors:** Michel Failing, Benchi Wang, Jan Theeuwes

**Affiliations:** 1 Department of Experimental and Applied Psychology, Vrije Universiteit Amsterdam, Van der Boechorststraat 1, 1081 BT Amsterdam, The Netherlands; 2 Institute of Brain and Behavior Amsterdam, Amsterdam, The Netherlands

**Keywords:** Selective, Attentional capture, Cognitive and attentional control

## Abstract

Where and what we attend to is not only determined by what we are currently looking for but also by what we have encountered in the past. Recent studies suggest that biasing the probability by which distractors appear at locations in visual space may lead to attentional suppression of high-probability distractor locations, which effectively reduces capture by a distractor but also impairs target selection at this location. However, in many of these studies introducing a high-probability distractor location was equivalent to increasing the probability of the target appearing in any of the other locations (i.e., the low-probability distractor locations). Here, we investigate an alternative interpretation of previous findings according to which attentional selection at high-probability distractor locations is not suppressed but selection at low-probability distractor locations is facilitated. In two visual search tasks, we found no evidence for this hypothesis: there was no evidence for spatial suppression when only target probabilities were biased (Experiment 1), nor did the spatial suppression disappear when only the distractor probabilities were biased while the target probabilities were equal (Experiment 2). We conclude that recurrent presentation of a distractor in a specific location leads to attentional suppression of that location through a mechanism that is unaffected by any regularities regarding the target position.

## Introduction

When interacting with the world, our visual system is constantly exposed to a vast amount of information. Attentional selection is the mechanism by which the visual system enables further processing of specific information at the cost of other information. Traditionally often defined as a dichotomous mechanism driven by (bottom-up) stimulus salience or (top-down) goals and intentions (e.g. Theeuwes, 2010; Wolfe & Gray, 2007), more recent evidence suggests that selection is also often driven by past episodes of selection (Awh, Belopolsky, & Theeuwes, 2012; Failing & Theeuwes, 2018; Theeuwes, 2019). It is typically assumed that selection priority due to these three mechanisms is represented on a shared priority map that is a topographical representation of selection priority (Failing & Theeuwes, 2018; Theeuwes, 2019). In this framework, moment-to-moment selection is determined by the highest activity on that map in a winner-takes-all fashion.

When investigating the influence of selection history, one typically observes that a location or stimulus feature that has been attended more frequently in the past is more likely to be selected again such that a stimulus at that location (or its feature) is either more efficiently selected when it is a target or is more distracting when it is a non-target. The idea, then, is that these past selection episodes have resulted in a stronger selection signal on the shared priority map. The influence of selection history on current selection has been demonstrated in its most basic form in research on (intertrial) priming (e.g., Maljkovic & Nakayama, 1994; Pinto, Olivers, & Theeuwes, 2005), but can also be observed due to, for example, reward (e.g., Anderson, Laurent, and Yantis, 2011; Failing & Theeuwes, 2014; Le Pelley, Pearson, Griffiths, & Beesley, 2015) or threat (e.g., Nissens, Failing, & Theeuwes, 2017; Schmidt, Belopolsky, & Theeuwes, 2015), and persists even when the previously selected stimulus becomes entirely task irrelevant and/or non-salient (for an extensive review see Failing & Theeuwes, 2018).

Recently, Wang and Theeuwes (2018a) provided evidence that, under certain circumstances, past episodes of selection may not necessarily lead to an increased likelihood of selection but instead may lead to a reduced likelihood of selection (see also Feldmann-Wüstefeld and Schubö, 2013; Ferrante et al., 2018; Goschy, Bakos, Müller, & Zehetleitner, 2014). In their experiments, participants performed the additional singleton task (Theeuwes, 1991, 1992) in which they had to search for a shape singleton (e.g., a circle among diamonds or vice versa) and identify the orientation of a line segment inside that shape singleton. On some trials, one of the non-target shapes was a color singleton (i.e., the additional singleton) that had to be ignored. The critical manipulation was that the distractor was more likely to appear in one specific location of the visual field. Replicating the traditional finding of attentional capture by physically salient stimuli, they observed that reaction times (RTs) were slower in distractor-present compared to distractor-absent trials. Importantly, though, RTs in distractor-present trials were significantly faster when the distractor appeared in its high-probability location compared to when it appeared in any of the other locations (i.e., the low-probability distractor locations). Moreover, they found that RTs in distractor-absent trials were slower whenever the target appeared in the high-probability distractor location. Wang and Theeuwes argued that the reduction in capture and the less efficient target selection was due to spatial suppression of the high-probability distractor location. Further supporting this notion was the observation of a suppression gradient: capture by the distractor was most attenuated at the high-probability distractor location while it became gradually stronger the further away the actual distractor location was from the high-probability distractor location. Interestingly, these effects seemed to be largely beyond the awareness of the participants as most of them failed to identify any regularities regarding the distractor position. Furthermore, Wang and Theeuwes (2018b) provided evidence that the same effect is not obtained when endogenously cueing probable distractor locations before the onset of the search display on a trial-by-trial basis. This underscores that spatial suppression is the consequence of “experiencing” past selection episodes rather than the result of an endogenous attempt to suppress a location in space.

As convincing as the conclusions drawn from these studies may be, spatial suppression of the high-probability distractor locations is but one of the possible explanations for the observed patterns. An equally plausible interpretation is that it is not the high-probability distractor location that is suppressed, but instead that selection of the target at the low-probability distractor locations is facilitated. In fact, since the distractor was more likely to appear at one location, the target was more likely to appear at any of the other locations (Wang & Theeuwes, 2018a, 2018b). More frequent selection of the target appearing in all locations except the high-probability distractor location may therefore have facilitated selection at these locations, possibly functionally akin to feature-related enhancements that are typically observed in a visual cortex (e.g., Bichot, Rossi, Desimone, 2005). In other words, because of an increase in activation for all locations except the high-probability location, target selection at these locations is facilitated and, if a distractor happens to be presented there, attentional capture is stronger than at the high-probability location. Such a mechanism can explain the entire pattern of results in Wang and Theeuwes (2018a, 2018b, 2018c).

The idea that repeated selection of a target at specific locations facilitates subsequent selection of targets presented in those locations fits very well with a whole range of findings. For example, two decades ago contextual cueing studies already showed that search targets that appeared in search display configurations that had already been searched before were more quickly found than targets that appeared in entirely new, unsearched display configurations (e.g., Chun & Jiang, 1998; Chun, 2000). Biasing target probabilities has since also been shown to lead to implicit attentional biases that are strong enough to overcome spatial neglect (Geng & Behrmann, 2005). Moreover, literature on selection history in the even context of intertrial priming (e.g., Maljkovic & Nakayama, 1994; Pinto et al., 2005) and rewarding stimuli (e.g., Anderson et al., 2011; Failing & Theeuwes, 2014) or threatening stimuli (e.g., Schmidt et al., 2015; Nissens, Failing, & Theeuwes, 2017) showed that previously selected stimuli are more likely to be selected again no matter whether they are currently the target or a distractor (cf. Della Libera & Chelazzi, 2009).

The present experiments test the hypothesis that the pattern of results in Wang and Theeuwes (2018a, 2018b, 2018c) is not the result of suppression of the high-probability distractor location but instead due to increased activation of the low-probability distractor locations (i.e., the high-probability target locations). To this end, we tested the two most extreme cases of this alternative explanation: In Experiment 1, only the probability of the target position was biased while the probability of the distractor was kept uniform, and in Experiment 2, only the probability of the distractor position was biased while the probability of the target was kept uniform. More specifically, participants had to perform the additional singleton task in both experiments. In the first experiment, the target was less likely to appear in one location than in all other locations while the distractor was presented in all locations with equal probability. If the previously observed spatial suppression of high-probability distractor locations is solely the result of the target being less probable to appear in that location, then we would expect to see the same results as in Wang and Theeuwes (i.e., less capture by the distractor when presented in a low-probability target location). If, however, the probability of the target is not the cause of the reduced attentional capture, we would expect that capture is equally strong regardless of where the distractor is being presented (i.e., the target probability manipulation has no bearing on the capture effect). In the second experiment, we manipulated the probabilities, such that the distractor was more likely to appear in one location while, crucially, the target was equally likely to appear in any location. To foreshadow the results, when only manipulating target probabilities, there was no spatially-specific suppression of attentional capture by the distractor, yet target selection was impaired for the low-probability target location (Experiment 1). In addition, when ensuring that the target was equally likely to appear in any location while the distractor was more likely to appear in a specific location, there was spatially-specific suppression of capture by the distractor as well as impaired target selection at the high-probability distractor location (Experiment 2).

## Experiment 1

### Methods

#### Participants

Twenty-four healthy adults (12 females, age *M*=21.1) with reported normal or corrected-to-normal vision who were naïve as to the purpose of the study participated in Experiment 1. The study was approved by the ethics committee of the Vrije Universiteit Amsterdam and informed consent was obtained before the experiment began. Participants received monetary compensation or course credits for their participation.

#### Apparatus and stimuli

Stimulus presentation and response registration were controlled by custom Python scripts. Stimuli were presented on a black background (~0 cd/m^2^) at a distance of 75 cm. A white fixation cross was visible throughout each trial. The search display consisted of eight discrete stimuli with different outline shapes in either one of two colors (red; RGB 255/0/0; green; RGB 0/255/0) and presented on an imaginary circle at equal distance (4° visual radius). These shapes were either one circle (1° radius) and seven diamonds (2° × 2°) or vice versa and contained a vertical or horizontal gray line segment (0.3° × 1.5°).

#### Procedure and design

Each trial consisted of a fixation display and a search display. After the fixation display, showing only the fixation cross for 1,000–1,250 ms, the search array was presented for 3,000 ms or until response. Participants were asked to covertly search for the shape singleton and indicate the orientation of the line segment inside that shape singleton as fast and as accurately as possible. Following timed-out or incorrect responses, a warning message was shown (“Response error – please maintain attention!”).

**Fig. 1:**
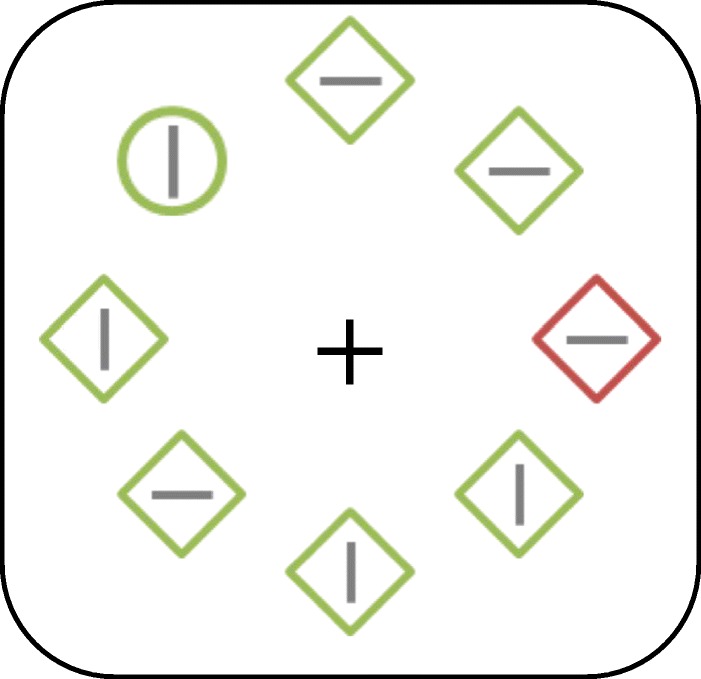
Example search display. Here, the target (shape singleton) is the green circle and the distractor (color singleton) is the red diamond. Participants are asked to indicate the orientation of the line segment inside the target

Two design features were important in this experiment. First, while all shapes were of the same color in one-third of the trials (distractor-absent trials), there was an additional singleton (“distractor”) present in the search display on the remaining two-thirds of trials (distractor-present trials; Fig. 1). This distractor was a color singleton (e.g., red among green or vice versa). Second, while the distractor was equally likely to occur at any location, the presentation of the target was systematically biased. Specifically, in distractor-present trials, the target never (i.e., in 0% of the distractor-present trials) appeared in one of the eight possible locations of the search display (fixed location; counterbalanced across participants). On distractor-absent trials, however, the target was equally likely to appear in any location (12.5%). In other words, this experiment had one low-probability target location in which the target appeared in ~4.2% of all trials in the experiment and seven high-probability target locations in which the target could appear in ~13.7% of all trials. The only constraint was that the target could never appear in the low-probability target location in distractor-present trials.

Each participant performed one practice block of 40 trials and six experimental blocks of 120 trials, yielding a total of 760 trials. All conditions were randomized within each block. After performing the search task, participants were asked to answer two questions. For the first question, they were required to indicate whether they were aware that the target was presented less often in one particular location. If they answered with “yes,” they had to indicate that location on an illustration of the search display. For the final question, they were asked to indicate the confidence about their answer on a 6-point scale (going from not confident at all, i.e., 0% sure, to very confident, i.e., 100% sure).

### Results

Trials in which participants did not respond in time as well as trials in which RTs were larger or smaller than 2.5 standard deviations from the average response time per block per participant were excluded from the analyses (around 4.5% of all data).

To evaluate the strength of the evidence for the alternative hypothesis (H1; i.e., condition difference) over the null hypothesis (H0; i.e., no condition difference) whenever a comparison using traditional null hypothesis testing was non-significant, we also quantified the Bayes factor (BF) using Bayesian hypothesis testing in JASP (JASP Team, 2018; Wagenmakers et al., 2017). In this framework a BF of 1–3 reflects anecdotal evidence, 3–10 moderate evidence, and >10 strong evidence in support of H1 (Wagenmakers et al., 2017). Likewise, a BF below 1 reflects quantification of evidence in support of H0 such that a BF of 1–.33 reflects anecdotal evidence, .33–.1 moderate evidence, and <.1 strong evidence in favour of the null hypothesis.

#### Attentional capture effect

**Fig. 2:**
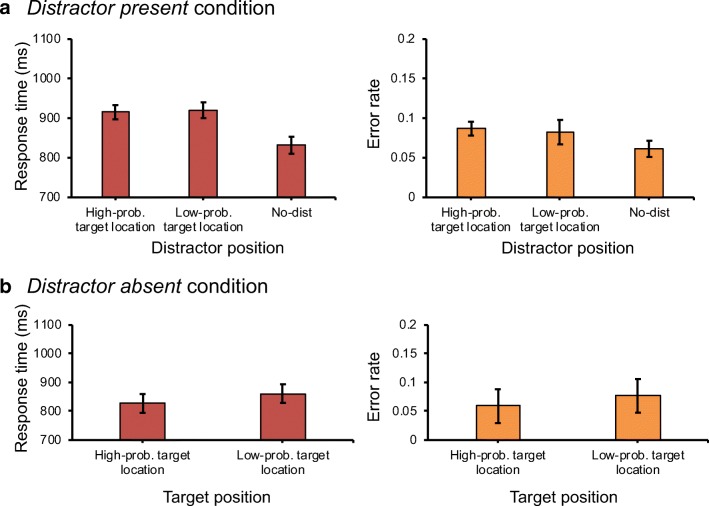
Results of Experiment 1. (**A**) The mean response times (left panel) and the mean error rates (right panel) as a function of the distractor position in distractor-present trials. (**B**) The mean response times (left panel) and the mean error rates (right panel) as a function of the target position in distractor-absent trials. Error bars here and in all other graphs represent 95% within-subject confidence intervals (Morey, 2008)

RT and error rate data of the distractor-present trials were first sorted according to the location of the distractor. An ANOVA on mean RT with distractor position (low-probability target location, high-probability target location, and distractor absent) as within-subject factor showed a main effect for distractor position, *F*(2,46)=38.94, *p*<.001, *ηp*^*2*^=.63. Planned comparisons revealed that, relative to when there was no distractor (*M*=832 ms), RTs were significantly slower when the distractor appeared in either the low-probability target location (915 ms), *t*(23)=7.25, *p*<.001, *d*=0.48, or the high-probability target location (920 ms), *t*(23)=7.48, *p*<.001, *d*=0.47. This indicates that the distractor captured attention. However, RT interference due to the presence of the distractor did not differ for when it was presented at either a low- (915 ms) or a high-probability (920 ms) target location, *t*(23)=0.48, *p*=.635, *d*=0.02, BF_10_=.239 (Fig. 2A, left panel), suggesting that the distractor captured attention equally strongly irrespective of where it appeared.

A similar analysis on error rate mimicked those of the RT analysis. The ANOVA on error rate with distractor position (low-probability target location, high-probability target location, and distractor absent) as within-subject factor showed a main effect for distractor position, *F*(2,46)=7.49, *p*=.002, *ηp*^*2*^=.25. Planned comparisons showed that the error rate was significantly lower in the distractor-absent trials (6.1%) compared to when the distractor appeared in either the low-probability target location (8.7%), *t*(23)=2.33, *p*<.001, *d*=0.21, or the high-probability target location (8.2%), *t*(23)=6.87, *p*<.001, *d*=0.2. There was no significant difference between trials in which the distractor appeared in the low-probability target location relative to when it appeared in the high-probability target location, *t*(23)=0.8, *p*=.541, *d*=0.04, BF_10_=.287 (Fig. 2A). This supports the RT findings and demonstrates that there was no speed-accuracy trade-off.

#### Target at low-probability target location

To determine whether biasing target presentation had any influence on the efficiency of target selection in the absence of the distractor, trials in the distractor-absent condition were sorted according to the target location. Note that only distractor-absent trials were analysed since the target never appeared in the low-probability target location during distractor-present trials. A paired-samples *t*-test on mean RT showed a significant difference between trials in which the target appeared in the low-probability target location (861 ms) relative to when it appeared in any of the other locations (827 ms), *t*(23)=2.13, *p*=.039, *d*=0.2 (Fig. 2B). A similar analysis on mean error rate revealed no significant difference (5.9% vs. 7.7%), *t*(23)=1.23, *p*=.231, *d*=0.16, BF_10_=.421 (Fig. 2B).

It is possible that the above-reported findings are not due to spatial regularities regarding the target position but instead due to the well-known phenomenon of inter-trial priming (e.g., Maljkovic & Nakayama, 1994). Following this notion, facilitated selection at high-probability target locations might be due to facilitated target selection in trials where the target position is the same as in the immediately preceding trial. To exclude this alternative explanation, we excluded all trials in which the location of the target repeated from one trial to the next. A paired-samples *t*-test on mean RT showed a significant difference between trials in which the target appeared in the low-probability target location (860 ms) relative to when it appeared in any of the other locations (827 ms), *t*(23)=2.16, *p*=.041, *d*=0.19. This shows that the effect on target selection is unaffected by intertrial priming.

To assess the spatial distribution of the interference in RT that was observed, distractor-absent trials were sorted according to the distance of the target position from the low-probability target location (with dist-0 being the low-probability target location and dist-5 the location on the opposite side of the imaginary circle). If the interference exhibits a spatial gradient that is similar to the previously observed distractor suppression gradient around a high-probability distractor location (e.g., Wang & Theeuwes, 2018a), one would expect that mean RT in this comparison reduces gradually with increasing distance from the low-probability target location. However, a repeated measures ANOVA on mean RT with distance (dist-0 through dist-5) as within-subject factor showed no significant main effect, *F*(4,92)=1.91, *p*=.115, *ηp*^*2*^=.08, BF_10_=.409. This suggests that instead of exhibiting a spatial gradient, interference in target selection during distractor-absent trials was highly localized to the low-probability target location.

#### Awareness assessment

Only two participants correctly identified the low-probability target location with a relatively low confidence (averaging 40%). The remaining participants were either unaware of any regularity (10 participants) and thus did not indicate any regularity or reported awareness of a regularity but indicated an incorrect regularity (12 participants).

### Discussion

Wang and Theeuwes (2018a, 2018b, 2018c) presented the singleton distractor much more often in one location than in all other locations, resulting in a reduced capture effect when the distractor appeared in this high-probability location relative to all other locations. However, because of this manipulation the target singleton was presented much less often in this location as well. While it is possible that presenting a distractor more often in one location led to the reduction in capture by the distractor in that location, as concluded by Wang and Theeuwes, it is also possible that presenting the target more often in the other locations may have increased capture by distractors appearing in these locations (i.e. low-probability distractor locations). In Experiment 1 we tested this alternative explanation by presenting the target much less often in one location than in all other locations, while the distractor was presented equally often in all locations. However, we found no reduced capture effect, indicating that the probability of where the target appears has no bearing on the processing of the distractor. Maybe unsurprisingly, we only found an influence of this manipulation on the processing of the target in distractor-absent trials: if the target happened to be present in a low-probability target location its selection was less efficient than when it was presented in a high-probability target location. This shows that participants learnt the probabilities of the target location and selection is biased accordingly (see Geng & Behrmann, 2005; Ferrante et al., 2018).

## Experiment 2

Experiment 1 in the context of the previous studies suggests that biasing the presentation of a salient distractor towards a specific location is necessary to induce spatial distractor suppression. However, it is still possible that such a manipulation alone is not sufficient for distractor suppression to occur. Indeed, it may be that only by combining a bias in target presentation away from one location and a bias in distractor presentation towards the same location, this location becomes suppressed such that both target selection is impaired and capture by the distractor is reduced at this location.

To investigate whether biasing distractor presentation alone is sufficient to elicit spatial suppression, we employed the same manipulation as Wang and Theeuwes (2018a). That is, the distractor was presented much more often in one location than in all other locations in Experiment 2. Crucially, however, we also ensured that the target was equally likely to appear in each location in both distractor-present and -absent trials. If presenting the distractor singleton much more often in one location than in all other locations results in a suppression of that location even when the target is equally likely to appear in any of the other locations, we would expect to find the same results as Wang and Theeuwes (2018a). If differences in the probability of presenting the target were critical for observing the reduced capture effect in the previous studies, we would expect no suppression of the location that is likely to contain the distractor.

### Methods

Another set of 24 healthy adults (14 females, age *M*=23.9) participated in Experiment 2. The stimuli, procedure, and experimental design of this experiment were similar to Experiment 1 except that we dropped the first awareness questions. That is, all participants had to indicate a regularity at the end of the experiment whether they reported to be aware of any or not.

Another, yet critical, difference to Experiment 1 was a change in the manipulation of target and distractor probabilities. In general, the conditions regarding the spatial position of the target and the distractor were more akin to Wang and Theeuwes (2018a) in that the distractor was more likely to appear in one specific location. In distractor-present trials of Experiment 2, the distractor appeared in 65% of the trials, or ~43.3% of all trials in the experiment, in one specific location (“high-probability distractor location”; counterbalanced across participants) and in 5% of the trials, or ~3.3% of all trials, in each of the seven remaining locations (“low-probability distractor locations”). Crucially different to Wang and Theeuwes (2018a), however, the target was equally likely to appear in any location in distractor-present as well as distractor-absent trials. That is, the target appeared in 12.5% of all distractor-present trials and 12.5% of all distractor-absent trials in any given location.

### Results

Applying the same data exclusion criteria as in Experiment 1 led to the exclusion of around 3.5% of the data from the analyses.

#### Attentional capture effect

**Fig. 3:**
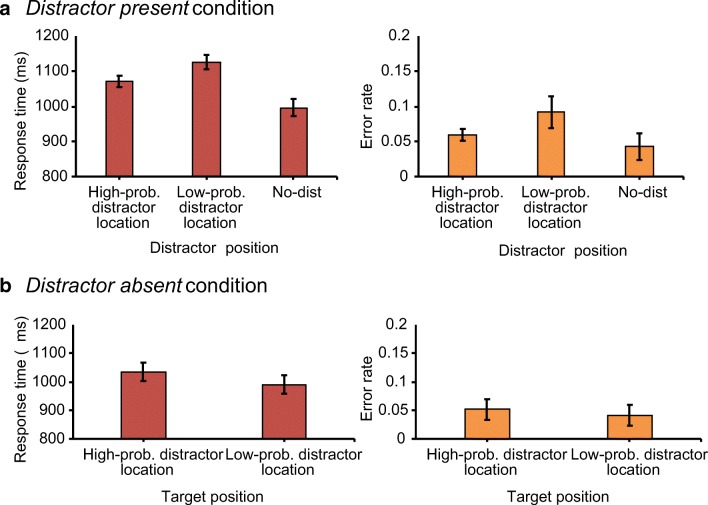
Results of Experiment 2. (**A**) The mean response times (left panel) and the mean error rates (right panel) as a function of the distractor position in distractor-present trials. (**B**) The mean response times (left panel) and the mean error rates (right panel) as a function of the target position in distractor-absent trials

In order to assess whether spatial suppression for a high-probability distractor location is observed when the target is equally likely to appear in each location, RT and error rate data were first sorted according to the location of the distractor. An ANOVA on mean RT with distractor position (high-probability distractor location, low-probability distractor location, and distractor absent) as within-subject factor showed a main effect for distractor position, *F*(2,46)=58.82, *p*<.001, *ηp*^*2*^=.72. Planned comparisons revealed that, relative to when there was no distractor (996 ms), RTs were significantly slower when the distractor appeared in either the high-probability distractor location (1,070 ms), *t*(23)=6.28, *p*<.001, *d*=0.23, or the low-probability distractor location (1,126 ms), *t*(23)=9.14, *p*<.001, *d*=0.23. This indicates attentional capture by the distractor. Importantly, and in line with findings from previous studies, interference in RT was significantly lower when the distractor appeared in the high-probability compared to when it appeared in the low-probability distractor location, *t*(23)=5.81, *p*<.001, *d*=0.17 (Fig. 3A). This shows that for this high-probability location attentional capture by the distractor was reduced, suggesting spatial suppression of the high-probability distractor location.

The results of the analysis on error rates resembled those on RT. The ANOVA on error rate with distractor position (high-probability distractor location, low-probability distractor location, and distractor absent) as within-subject factor showed a main effect for distractor, *F*(2,46)=12.5, *p*<.001, *ηp*^*2*^=.35. Planned comparisons showed that error rate was higher when there was a distractor present in either the high-probability distractor location (5.9%), *t*(23)=2.81, *p*=.01, *d*=0.43, or any of the low-probability distractor locations (9.2%), *t*(23)=3.7, *p*=.001, *d*=0.3, relative to when there was no distractor present (4.3%). Importantly, error rate was significantly lower for when the distractor appeared in the high-probability distractor location relative to the low-probability locations, *t*(23)=3.46, *p*=.002, *d*=0.69 (Fig. 3A). This indicates that there were no speed-accuracy trade-offs.

**Fig. 4:**
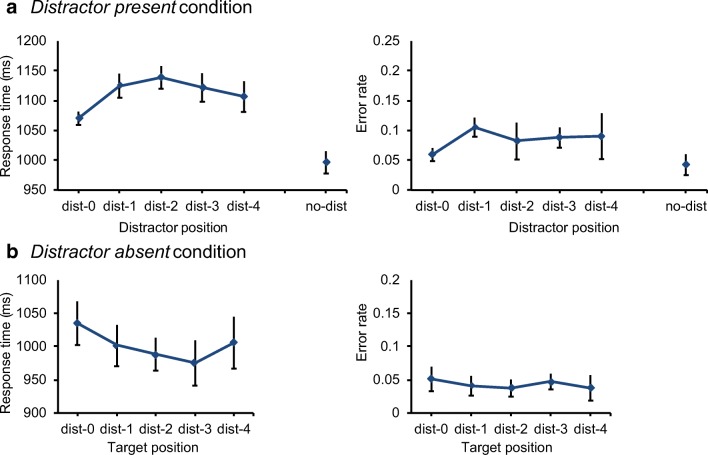
Results of Experiment 2. The spatial distribution of the interference in target selection as a function of the distractor position in distractor-present trials (**A**) and as a function of target position in distractor-absent trials (**B**). Mean response times are shown in the left panels and mean error rates are shown in the right panels. *Dist-0* refers to the high-probability distractor location, *dist-1* refers to the low-probability distractor location right next to the high-probability distractor location (left and right; 45° polar angle), and so on

To examine the spatial distribution of the reduction in distractor interference as was done in the previous reports on distractor suppression (e.g., Wang & Theeuwes, 2018a, 2018b, 2018c), RT was examined after sorting data according to the distance of the distractor to the high-probability distractor location (with dist-0 being the high-probability distractor location and dist-5 being the location on the opposite side of the imaginary circle). A repeated measures ANOVA showed a significant main effect for distance, *F*(4,92)=4.08, *p*=.004, *ηp*^*2*^=.15. Figure 4A (left panel) shows that RT plateaued at the dist-2 location. To describe the nature of the trend, a linear function was fitted for the data from dist-0, i.e., the high-probability distractor location, to dist-2, the plateau of the distribution. The slope (28.38 ms per point of distance) was significantly larger than zero, *t*(23)=6.0, *p*<.001, *d*=1.57, which shows that the reduction in distractor interference became smaller with increasing distance of the location of the distractor from its high-probability location. Identical analyses on error rates revealed similar results. The repeated measures ANOVA showed a significant main effect of distance, *F*(4,92)=2.83, *p*=.029, *ηp*^*2*^=.11, and the slope of the linear function (1.22% per display element) was marginally larger than zero (Fig. 4A, right panel), *t*(23)=1.86, *p*=.075, *d*=0.57. *Post hoc* inspection of the gradient suggests that the pattern observed here might not be best described by a linear function. Nonetheless, in line with previous studies, there is convincing evidence for a spatial gradient of suppression.

We also assessed the possibility that inter-trial location priming drove the suppression (e.g., Maljkovic & Nakayama, 1994). Indeed, the location of the distractor could repeat from one trial to another allowing for location priming to occur. To exclude this alternative explanation, we excluded all trials in which the location of the distractor repeated from one trial to the next. The ANOVA on mean RTs with distractor position (high-probability distractor location, low-probability distractor location, and distractor absent) as within-subject factor showed a main effect, *F*(2, 46)=48.05, *p* < .001, *ηp*^*2*^=.68. Relative to when there was no distractor (996 ms), RTs were significantly slower when the distractor appeared in either the high-probability distractor location (1,082 ms), *t*(23)=6.25, *p*<.001, *d*=0.26, or the low-probability distractor location (1,126 ms), *t*(23)=9.14, *p*<.001, *d*=0.27. Crucially, and in line with previous findings, interference by the distractor was reduced whenever it appeared in the high-probability location, *t*(23)=3.45, *p*=.002, *d*=0.13. This indicates that the previously reported effect is not the consequence of inter-trial location priming.

#### Target at the high-probability distractor location

To determine the efficiency of target selection in the absence of a distractor, we first analysed RT in distractor-absent trials. A paired-samples *t*-test showed that participants were significantly slower when the target appeared in the high-probability distractor location (1,035 ms) relative to when it appeared in one of the low-probability distractor locations (991 ms), *t*(23)=2.67, *p*=.013, *d*=0.14 (Fig. 3B). There was no effect on error rates (5.1% vs. 4.1%), *t*(23)=1.23, *p*=.232, *d*=0.21 (Fig. 3B).

To assess the spatial distribution of this interference in RT, distractor-absent trials were sorted according to distance of the target position to the high-probability distractor location (as was done for the other analyses). The repeated measures ANOVA on distance (dist-0 through dist-5) showed a marginally significant main effect, *F*(4,92)=2.39, *p*=.056, *ηp*^*2*^=.09, which plateaued at the dist-2 location (Fig. 4B). Fitting a linear function for the data on the first three locations revealed that the gradient in the distractor-absent condition was reversed relative to the one in the distractor-present condition. Interference reduced gradually with increasing distance of the target location to the high-probability distractor location. The slope was significantly different from zero (-21.83 ms per display element), *t*(23)=3.08, *p*=.005, *d*=0.92. There was no effect on error rates, *t*<1 (Fig. 4B).

In Experiment 2, the target could also appear in the high-probability distractor location in distractor-present trials. We therefore compared trials in which the target appeared in the high-probability distractor location with trials in which it appeared in any of the low-probability distractor locations. In line with the notion of spatial suppression of high-probability distractor locations, a paired-samples *t*-test showed that RT was significantly slower when the target appeared in the high-probability distractor location (1,134 ms vs. 1,083 ms), *t*(23)=3.63, *p*=.001, *d*=0.15. There were no significant differences in error rate.

#### Awareness assessment

Only nine participants correctly identified the high-probability distractor location with a relatively low average confidence (31.1% sure). The remaining participants (i.e., 15 participants) indicated an incorrect regularity. Since more participants correctly identified the high-probability location than would be expected by chance, we analyzed whether awareness had a significant effect on the suppression effect. However, an ANOVA on mean RT with awareness (aware vs. unaware) and distractor position (high-probability distractor location, low-probability distractor location, and distractor absent) as factors showed no significant interaction between awareness and distractor position, *F*(2,44)=.21, *p=*.808, *ηp*^*2*^=.09. To quantify the evidence against the interaction effect, we compared the model with both main effects against the model with the main effects and the interaction term. This comparison showed moderate evidence against the model that included the interaction (BF = 3.79). Analogously, comparing the model with the interaction against all other models provided evidence against the interaction (BF_m_ = .473). In other words, there was no evidence that awareness affected suppression.

### Discussion

The current experiment replicates all findings of Wang and Theeuwes (2018a, 2018b), even when the target is equally likely to appear in all locations. As in Wang and Theeuwes (2018a, 2018b), we showed reduced capture by the singleton distractor when it appeared in the high-probability distractor location and less efficient selection when the target happened to be presented in that location. Moreover, we found a spatial gradient effect of suppression around that location, evident both in distractor suppression and in less efficient selection of the target.

## General discussion

The present study provides confirmatory evidence for selective suppression of a location that is more likely to contain a singleton distractor than all other locations (Wang & Theeuwes, 2018a, 2018b, 2018c). Our Experiment 1 shows that those results cannot be explained by assuming that, instead of suppression of high-probability distractor locations, participants prioritize locations where the target is more likely to appear (i.e., the low-probability distractor locations). Similarly, Experiment 2 shows selective suppression of the location that is likely to contain a distractor singleton even when each location is equally likely to contain a target singleton. The conclusion is that by manipulating the probability of where the distractor is likely to appear, participants learn to suppress the location that is most likely to contain a distractor and that this occurs independently of the probability of where the target singleton appears.

This conclusion should be taken in the context of the current study in which a salient distractor is present because – in general – manipulating the probability of where the target is likely to appear will most certainly bias attention. For example, the classic cueing studies of Posner (1980) have shown that people are faster to detect targets appearing in probable locations than improbable locations (Shaw and Shaw, 1977). Similarly, contextual cueing studies show that targets that are repeatedly searched in the same visual search displays are found more quickly compared to when people search new, unseen search displays (e.g., Chun & Jiang, 1998; Chun, 2000). Ferrante et al. (2018) showed a bias towards locations that were most likely to contain a target (see also Geng & Behrmann, 2005). Also, our Experiment 1 shows this effect: in the distractor-absent condition, if the target singleton happened to be in the low-probability location, its selection was less efficient than when it was presented in a high-probability location, which indicates that participants have learned the probabilities of the target location and selection was biased accordingly. Importantly though, this bias did not result in a reduction of capture by the distractor singleton. More specifically, our two experiments demonstrate that manipulating the probability of where a target is likely to appear is neither a necessary nor a sufficient means to induce suppression of a salient distractor.

Although there was no evidence that would support an alternative explanation of the previously reported distractor suppression effects (Wang & Theeuwes, 2018a, 2018b, 2018c) in terms of a bias in the target location probabilities, it is still interesting to speculate about the target probability effect we observed in Experiment 1. It might be that the impaired selection for targets appearing in the low-probability target location during distractor-absent trials reflects a form of spatial suppression of that location. Alternatively, it might not be an impairment of target selection at that location, but a facilitation of target selection at high-probability target locations. The fact that this effect had no bearing on distractor processing during distractor-present trials shows that whichever mechanism it may be, it is likely to be driven by a combination of spatial and feature-specific information of the target. This is in line with very recent evidence suggesting that attentional biases due to statistical regularities rely on the regularities of information from both space and feature dimensions (e.g., color or orientation; Failing, Feldmann-Wüstefeld, Wang, Olivers, & Theeuwes, 2019; Stilwell, Bahle, & Vecera, 2018). Future studies will be necessary to provide a clear characterization of the underlying mechanism of this effect.

Our results regarding the awareness of the statistical regularities in the display are in line with previous studies in which spatial probabilities regarding targets and/or distractors were manipulated. Experiment 1 shows that although the behavior of the participants was affected by our manipulation of target probabilities, the vast majority of participants did not become explicitly aware of this spatial regularity (see also Chun & Jiang, 2003; Geng & Behrmann, 2005). Similarly, participants in Experiment 2 were largely not able to indicate the correct regularity regarding the presentation of the salient distractor although the regularity affected their behavior systematically (see also Failing et al., 2019; Wang & Theeuwes, 2018a, 2018b). While it is noteworthy that, in Experiment 2, there were more participants aware of the regularity than would be expected by chance, there was no evidence that the spatial suppression effect differed among those who were and those who were not aware. Together, this provides support for the notion that observers learn statistical regularities in the visual environment, which then selectively bias attention even though they have little awareness for what they have learned.

In sum, the current study confirms that statistical regularities regarding the location of a distractor singleton will bias attention. We assume that extracting these spatial statistical regularities creates a priority signal that feeds into the spatial priority map such that locations that are likely to contain a distractor competes less for attention than all other locations.
